# Cerebro-Spinal Meningitis

**Published:** 1870-10

**Authors:** J. E. Harvey

**Affiliations:** Walkerville, Ill.


ARTICLE VII.—*Cerebro-spinal Meningitis and Chloroform.* By J. E. HARVEY, M.D., Walkerville, Ill.

Having read the case of trismus nascentium cured by the application of chloroform to the spine, as reported by T. L. Papin, M.D., of St. Louis, it occurred to me that it would be equally efficacious in cerebro-spinal meningitis.

On the 12th of August last, I was called to a case of the above-named disease, occurring in a boy two and a half years of age. I applied sulphuric ether (having no chloroform), by means of folds of flannel wet with it and placed along the spine, from the occiput to the sacrum; and gave it by inhalation at the same time, though not to the extent of producing insensibility. The effect was most gratifying, overcoming in three hours the condition of tonic contraction of the extensor muscles of the spine and the flexors of the legs; on the omission of the application, the muscles became again rigid on two occasions, but soon yielded; the patient was convalescent upon the fifth day from the attack. The patient was kept upon the muriated tincture of iron throughout the attack.

